# Nanomaterials–plants–microbes interaction: plant growth promotion and stress mitigation

**DOI:** 10.3389/fmicb.2024.1516794

**Published:** 2025-01-15

**Authors:** Gurleen Kaur Sodhi, Tharuka Wijesekara, Kailash Chand Kumawat, Priyanka Adhikari, Kuldeep Joshi, Smriti Singh, Beatrice Farda, Rihab Djebaili, Enrico Sabbi, Fares Ramila, Devendra Sillu, Gustavo Santoyo, Sergio de los Santos-Villalobos, Ajay Kumar, Marika Pellegrini, Debasis Mitra

**Affiliations:** ^1^University Institute of Biotechnology, Chandigarh University, Mohali, Punjab, India; ^2^Department of Food Science and Agricultural Chemistry, Faculty of Agricultural and Environmental Sciences, McGill University, Sainte-Anne-de-Bellevue, QC, Canada; ^3^Department of Industrial Microbiology, Jacob Institute of Biotechnology and Bioengineering, Sam Higginbottom University of Agriculture, Technology and Sciences (SHUATS), Prayagraj, Uttar Pradesh, India; ^4^Central Ayurveda Research Institute, Kolkata, West Bengal, India; ^5^Centre for GMP Extraction Facility, National Institute of Pharmaceutical Education and Research, Guwahati, Assam, India; ^6^Department of Anaesthesia and Operation Theatre Technology, College of Pharmacy, Chandigarh Group of Colleges Jhanjeri (Mohali), Sahibzada Ajit Singh Nagar, Punjab, India; ^7^Department of Life, Health and Environmental Sciences, University of L’Aquila, L’Aquila, Italy; ^8^Laboratory Biotechnology, Water, Environment and Health, Abbes Laghrour University of Khenchela, Khenchela, Algeria; ^9^Laboratory of Mycology, Biotechnology and Microbial Activity, Brothers Mentouri University of Constantine 1, Constantine, Algeria; ^10^Department of Environmental Science and Engineering, Guangdong-Technion Israel Institute of Technology, Shantou, China; ^11^Instituto de Investigaciones Químico Biológicas, Universidad Michoacana de San Nicolás de Hidalgo, Ciudad Universitaria, Morelia, Michoacán, Mexico; ^12^Instituto Tecnológico de Sonora, Ciudad Obregón, Sonora, Mexico; ^13^Department of Microbiology, Graphic Era (Deemed to be University), Dehradun, Uttarakhand, India

**Keywords:** nanoparticles, nanotechnology, abiotic stress, biotic stress, sustainable agriculture

## Abstract

Soil salinization, extreme climate conditions, and phytopathogens are abiotic and biotic stressors that remarkably reduce agricultural productivity. Recently, nanomaterials have gained attention as effective agents for agricultural applications to mitigate such stresses. This review aims to critically appraise the available literature on interactions involving nanomaterials, plants, and microorganisms. This review explores the role of nanomaterials in enhancing plant growth and mitigating biotic and abiotic stresses. These materials can be synthesized by microbes, plants, and algae, and they can be applied as fertilizers and stress amelioration agents. Nanomaterials facilitate nutrient uptake, improve water retention, and enhance the efficiency of active ingredient delivery. Nanomaterials strengthen plant antioxidant systems, regulate photosynthesis, and stabilize hormonal pathways. Concurrently, their antimicrobial and protective properties provide resilience against biotic stressors, including pathogens and pests, by promoting plant immune responses and optimizing microbial-plant symbiosis. The synergistic interactions of nanomaterials with beneficial microorganisms optimize plant growth under stress conditions. These materials also serve as carriers of nutrients, growth regulators, and pesticides, thus acting like “smart fertilizers. While nanotechnology offers great promise, addressing potential environmental and ecotoxicological risks associated with their use is necessary. This review outlines pathways for leveraging nanotechnology to achieve resilient, sustainable, and climate-smart agricultural systems by integrating molecular insights and practical applications.

## 1 Introduction

The rapidly changing climatic conditions have introduced various factors that adversely impact the growth and productivity of agriculture and significantly affect the ability to meet food needs sustainably (Goud et al., 2022). Challenges are compounded by dependence on traditional methods, such as pesticides and growth-enhancing chemicals, which health experts frown upon due to their direct impacts on public health. Therefore, modern agriculture has an uphill task of maintaining crop productivity and sustainability because of the increasing occurrence of both biotic and abiotic stresses (Guntukula, 2020). These complex problems need novel solutions that balance effective crop management with environmental and consumer health considerations. Biological and environmental stressors substantially impact crop yields and plant growth, posing severe problems for ecosystems and agriculture (Kumari et al., 2022). Abiotic stress (e.g., drought, elevated temperatures, salinity, and toxic metals) is one of the most critical environmental variables influencing plant development and yield. This stress induces disturbances in physiological and metabolic functions in plants. In addition to abiotic stress, several biological stresses are induced by weeds, bacteria, viruses, fungi, and insects. Their adverse effects induce physical damage and infections in plants, decreasing crop productivity (Nnadi and Carter, 2021). Exploring innovative approaches to the synergistic relationships among microorganisms, nanomaterials, and plants could mitigate different stresses and plant growth. This three-way interaction contributes to plant health enhancement and offers a promising strategy for ensuring sustainability in agricultural practices (Campos et al., 2023).

Complex associations between microorganisms and plants form the basis for productivity and health in plant species. The rhizosphere of plants is colonized by symbiotic organisms, which include bacteria and fungi, which play a central role in nutrient uptake, growth promotion, and disease resistance in plants. The metabolites produced from these microorganisms are bioactive phytohormones that regulate plant growth and its responses to various abiotic stresses (Sodhi and Saxena, 2023). It has been observed that adding endophyte bacteria to stressed plants increases yield and overall development. Researchers are always looking for effective and sustainable ways to lessen biotic and abiotic pressures on plants (Pang et al., 2020). Promising strategies for handling both forms of stress can be found in nanotechnology. Nanomaterials can be developed with specific physicochemical properties to increase agricultural yields while decreasing inputs like fertilizers, insecticides, and growth regulators. They reduce environmental impact, enhancing efficiency in delivering active ingredients of such inputs and providing controlled release mechanisms (El-Saadony et al., 2022). It has been reported that many nanomaterials, like nanoparticles (NPs), nanotubes, and nanocomposites, act positively in soil nutrient availability and water-holding capacity. They also regulate plant development hormones, alleviating harm from severe environmental circumstances (Pramanik et al., 2023). Even if they can have some disadvantages, their mode of operation may complement the other methods in enhancing the plants’ resistance and yield, ensuring better sustainability of agriculture (Rajput et al., 2021; Hayat et al., 2023).

The synergy among plants, microorganisms, and nanomaterials could significantly increase plant stress mitigation. This relationship enhances plant nutrient uptake and utilization, improves defense strategies, reduces stress, and improves direct delivery systems (Azameti and Imoro, 2023). Simultaneously, these beneficial microbes fine-tune plant hormonal balance and elevate the production of various phytochemicals concerned with mechanisms against stress. Nanomaterials facilitate water retention and reduce plant oxidative damage under stress conditions. Consequently, beneficial microorganisms enhance the hydraulic conductivity of roots and facilitate osmotic adjustment, subsequently increasing plant tolerance to water scarcity. Additionally, nanomaterials can more efficiently transport microbial inoculants to the roots of plants, thus promoting effective rhizosphere colonization by these beneficial microbes (Dilnawaz et al., 2023). This is attained by continuous benefits being rendered and reducing the decline in microbial effectiveness, providing a practical framework for improving the durability and yield of plants under environmental stressors (Mohan et al., 2023).

Despite all the benefits associated with nanomaterials for use in agricultural activities, there are concerns regarding their limitations and hazards. Among the significant issues on this path are bioaccumulation and nanoparticles’ adverse effects on plant organisms. Owing to their diminutive dimensions, these materials can readily penetrate plant cells, influencing their physical properties and interfering with numerous physiological and biochemical processes (Gowtham et al., 2024). Research indicates that exposure to nanoparticles may result in disruptions at the molecular level, affecting enzymatic functions, photosynthesis, and root development, thereby ultimately diminishing agricultural productivity. Furthermore, nanoparticles may accumulate in terrestrial and aquatic systems and, therefore, pose a threat to ecosystems nearby and human health through the food chain as these particles are absorbed by crops and then consumed by humans (Murali et al., 2022). The lack of standardized evaluation methodologies and the need for long-term research into these chemicals’ impact makes the concerns over their impact on the environment and non-target organisms more alarming. Therefore, nanomaterials might benefit particular agricultural uses, but they require full regulation and a better understanding of their ecotoxicological impacts (Gottardo et al., 2021). Although many reviews have focused on the roles of nanomaterials in agriculture and plant stress responses, the current review has a different emphasis on new developments related to high-performance platforms and new applications of nanomaterials.

## 2 Factors affecting plant growth

Solar radiation is a very important factor for plant growth. However, too much radiation creates different types of abiotic stresses and exceptionally high temperatures that can harm photosynthesis and increase plants’ transpiration rate (Kosobryukhov et al., 2020; Durand et al., 2021). Excessively high or low temperatures adversely affect plants by causing the denaturation of proteins and interfering with cellular functions (Ul Hassan et al., 2022; Xu et al., 2022). Reduced temperatures impede biological processes, whereas elevated temperatures enhance them, resulting in imbalances that surpass the thresholds of heat shock proteins, which play a vital role in plant defense mechanisms (Moore et al., 2021; Sahil Keshan et al., 2021). Besides, enhanced UV-B radiation generates reactive oxygen species, which, in turn, enhances oxidative stress in plant systems (Shi et al., 2022; Tan et al., 2023). Drought stress is considered another important abiotic factor, which, according to some studies, affects the morphological aspects of plant roots by making the primary roots shallow and necessitating better soil stabilization (Iqbal M. S. et al., 2020; Lozano et al., 2020). Drought conditions lead to cellular dehydration, diminished stomatal conductance, and reduced carbon dioxide uptake (Ding et al., 2021; Seleiman et al., 2021). At the same time, saline conditions primarily aggravated by drought pose further stress to plants due to impaired ionic balance (Kaiwen et al., 2020; Stavi et al., 2021). In contrast, flooding reduces the rates of oxygen and carbon dioxide, which, in turn, will lead plants to conduct anaerobic metabolic activities, leading to toxic by-products toxic to cellular life processes (Merz et al., 2021). On the other side, heavy metals from human activities such as pesticide and fertilizer abuse induce plant stress (Kaur et al., 2020), resulting in symptoms such as leaf browning, chlorosis, and abnormalities in root structure (Kiran and Sharma, 2022).

### 2.1 Biotic stressor and their impact on plant growth

#### 2.1.1 Fungal diseases

The main biotic elements that affect the health and productivity of plants are fungal pathogens. Infection by fungi in plants can occur through lesions, natural openings such as stomata, or highly specialized structures called hyphae. These microbes then proliferate within the plant tissues to cause stunted growth and wilting and very often show as powder or rust-like lesions on foliage or stems (Fones et al., 2020; Arya et al., 2021). The most common phytopathogenic fungi, *Fusarium oxysporum*, and *Rhizoctonia solani* interfere with agricultural yield by interfering with the normal physiological functions of the plant. These may be quantified as a reduction in photosynthetic activities and nutritional uptake. Various substitutes have been developed to find new avenues for combating fungal diseases, incorporating nanotechnology-based approaches besides biological control. It has been reported that biosynthesis of selenium nanoparticles mediated through *Bacillus megaterium* exhibited very prominent activity against *Rhizoctonia solani* in faba bean. In its mode of action, ROS generation alters the cell wall topography of fungi and intracellular proteins and ultimately leads to cell death. Some works have identified enhancing resistance by SeNPs via an induced response in producing PR proteins, improving plant immunity (Hashem et al., 2022). Another approach via nanotechnology was the employment of ZnONPs, which also demonstrated very potential activity against fusarium wilt in eggplants. Accordingly, ZnO NPs alter the integrity of the fungus membrane, increase ROS accumulation, and eventually inhibit fungal growth. In addition to their antifungal effects, ZnO nanoparticles have the added advantage of enhancing plant growth by improving nutrient acquisition and facilitating photosynthesis (Abdelaziz et al., 2022). Other than nanoparticles, even plant growth-promoting fungi have been found effective as biocontrol agents. The hydrolytic enzymes produced by PGPF, including chitinases and glucanases, have long been recognized as degrading fungal cell walls and inhibiting their growth. These PGPFs were reported to effectively suppress disease incidence, promote growth, and improve health in tomato plants under a series of laboratory and greenhouse experiments conducted in the face of bacterial wilt, as reported by Attia et al. (2023).

#### 2.1.2 Bacterial diseases

Bacterial diseases in plants are brought about by invading plant tissues using pathogenic bacteria that produce substances injurious to plants and degrading cellular wall components, leading to cell death and wilting. The pathogenic bacteria, such as *Pseudomonas syringae* and *Xanthomonas campestris*, enter the plant through natural openings or wounds and colonize the vascular tissues, interfering with the transportation of nutrients and water within the plant as stated by Catara and Bella (2020). Consequently, the ensuing wilting, chlorosis, and necrosis markedly diminish crop yields. Among the numerous approaches attempted and proven quite effective, the application of plant growth-promoting rhizobacteria is included. These valuable microorganisms occupy an ecological niche in the rhizosphere, competing with phytopathogens for nutrition and space, decreasing their populations. PGPR may also produce several antimicrobial metabolites, such as siderophores, hydrolytic enzymes, and antibiotics, which interfere with the growth cycle of phytopathogenic bacterial species. Further, they induce systemic resistance and reinforce the immunity of the plant against infection with bacteria (Abdelaziz et al., 2023b).

#### 2.1.3 Viral diseases

Generally, the most challenging diseases to control are those caused by viruses, concerning their mode of infection and transmission in nature. The majority of plant viruses, including ToMV and CMV, are transmitted by insect vectors through the transmission with aphid, whitefly, and thrip vectors. After entering a susceptible host plant, viruses hijack the machinery of the host cells and force the host cells to synthesize viral proteins and RNA. The virus particles disseminate in the plant through its vascular tissues-xylem and phloem-through tiny openings called plasmodesmata (Trebicki, 2020; Jones, 2021). Symptoms of the viral infection include leaf curl, mosaic patterns on leaves, stunted growth, and reduction of the crop yield of the plant. New evidence shows that Se and nano-Se help plants cope with the virus more effectively. SeNPs enhance the activities of antioxidant enzymes, including SOD and CAT. These enzymes reduce oxidative stress, which increases during viral infection. Reducing oxidative stress strengthens the plant’s defense mechanisms, reducing the spread and replication of viruses. There is not enough direct evidence regarding the antiviral potential of SeNPs; however, they appear to exhibit a good resistance against viral infection in plants (Hashem et al., 2022).

#### 2.1.4 Nematodes or insects

Nematodes and insects are the two major agents of biotic stress that negatively affect crop productivity. Such herbivores become damaging agents to crops because they feed on plants. Root-knot nematodes (*Meloidogyne* spp.) are roundworms that cause the formation of galls on plant roots, thereby impeding the uptake of nutrients and water. Insects such as aphids, mites, and whiteflies can sap from the host plant tissue, suppress its vigor, and act as viral disease vectors (Secretariat et al., 2021). Chitosan- and EDTA-conjugated graphene oxide nanocomposites have been devised to combat nematode infections. The graphene oxide-chitosan conjugate possesses more significant nematicidal properties since chitosan is a naturally obtained biopolymer with known antimicrobial properties. The application of this composite, which hinders the movement and feeding processes of nematodes, was attributed to the significant reduction of nematode infection in eggplants. Further, the nanocomposite triggers plant immunity by expressing defense enzymes due to nematode attacks (Attia et al., 2022). The use of PGPF, along with fosthiazate, gives encouraging results against the control of root-knot nematodes in tomato plants. The fungi colonize the plant’s root system, and the fosthiazate nematicide shows a synergistic effect in inhibiting nematode reproduction, hence also enhances plant defense responses against the invading pest (Kandil et al., 2024). Nanotechnology-based approaches continue to devise methods for pest-insect control strategies.

#### 2.1.5 Weeds

Weeds are unwanted flora that proliferate in agricultural settings, unlike pests and illnesses. They often contend with indigenous flora for water, nutrients, and space. Certain weeds outcompete native plants, allowing them to access sunlight. The ability of native plants to perform photosynthesis is consequently diminished. Adjacent plants vie with weeds for moisture. This induces Extreme water stress, especially in regions susceptible to drought (Vilà et al., 2021). Allelochemicals are recognized to be emitted by several weed species. These allelopathic compounds inhibit plant growth, germination, and several physiological processes. Furthermore, it induces stress in plants, leading them to allocate resources toward regulating hormone signaling and producing stress-responsive genes. Furthermore, they secrete a range of lytic enzymes from their roots (Sharma et al., 2023).

## 3 Nanoparticles and their synthesis

### 3.1 Nanomaterials

Over the last couple of decades, the heavy use of chemical fertilizers and pesticides to achieve the desired crop output has adversely affected the soil’s natural attributes and caused considerable damage to the ecosystems (Alengebawy et al., 2021). Suitable alternatives are being sought which would not be detrimental to the environment. Nanotechnology has emerged as one of the feasible approaches offering new solutions to age-old problems with less environmental impact (Campaña et al., 2023; Nandini et al., 2023). Moreover, researchers have focused on using nanomaterials in agricultural practices due to existing climatic catastrophes, soil degradation, and agricultural land reduction out of the fear that meeting the future global demand for food would not be possible (Nandini et al., 2023).

Recent progress within nanotechnology has opened new horizons for mitigating plant stresses (Ijaz et al., 2023). Engineered nanomaterials with defined physico-chemical properties and a multi-functional capability, therefore, have great potential for improved performance and productivity of plants under suboptimal environmental conditions (Sun et al., 2022). Materials possessing structural components in at least one dimension within the 1–100 nanometers range are called nanomaterials (Boholm and Arvidsson, 2016). At the nanoscale, they exhibit unique physical and chemical properties, distinguishing them from their bulk equivalents (Baig et al., 2021). They have flexible electrical and mechanical properties, a high surface-to-volume ratio, and extraordinary chemical activity. Nanotechnology has led to rapid advances in many fields, such as material science and biomedical ones. One continually growing field is nanomaterial fabrication with two important preparation methodologies, namely, top-down and bottom-up syntheses (Singh et al., 2020; El-Khawaga et al., 2023; Mekuye and Abera, 2023). In the top-down method, a bulk material is first divided into smaller pieces to create nanomaterials. Standard top-down techniques include sputtering, chemical etching, thermal or laser ablation, mechanical grinding, and explosives. The bottom-up approach originates with atomic or molecular entities that aggregate to form nanomaterials. This method employs atomic or molecular condensation, vapor deposition, sol–gel processes, spray pyrolysis, chemical or electrochemical deposition, aerosol processes, and bioreduction (Abid et al., 2022). Based on their place of origin, NPs made with these techniques can be roughly categorized as organic, inorganic, polymeric, or hybrid nanomaterials. Based on material used they can be further categorized (Table 1). Biological processes or characteristics may be modified by applying recently engineered nanomaterials (Ijaz et al., 2023).

**Table 1:** Nanomaterials types.

Nanomaterials	Example
Metal	Copper, silver, titanium, and iron NPs
Metal oxide	Copper oxide, zinc oxide, iron oxide, titanium oxide, and magnesium oxide NPs
Polymeric	Micelles and chitosan NPs
Other	Nanosilica, silicon-based NPs, selenium NPs, carbon nanotubes, and graphene oxide

A variety of analytic techniques, such as X-ray diffraction (XRD), Scanning Electron Microscopy (SEM), Transmission Electron Microscopy (TEM), and Fourier Transform Infrared Spectroscopy (FTIR), are utilized to characterize the morphology, uniformity, and interactions of the newly synthesized NPs (Campaña et al., 2023).

### 3.2 Synthesis

Traditionally, the two most used methods mainly applied for the production of a higher yield of nanomaterials for various applications, such as in medicine, pharmaceutical industries, and agriculture for plant and soil health enhancement purposes, including chemical syntheses, such as silver nanoparticles and gold nanoparticles, and other metal nanoparticles via liquid phase. The other methods are microbial synthesis and fermentative processes, mainly plant pieces and soil. Production of nanomaterials via these chemical processes is relatively costly and has some environmental toxicity. However, eco-friendly and low-cost microbial synthetic methods used in producing metal nanoparticles are less efficient. Moreover, using these processes, it is relatively complex to produce the desired nanomaterials with the required targeted properties to meet the specific applications in agriculture (Bahrulolum et al., 2021; Saravanan et al., 2022). Although chemical methods are of superior efficacy and are heavily used in producing different nanomaterials with well-controlled properties, they are generally high-cost and may be toxic to the environment. Microbial synthesis of silver and gold nanoparticles is environmentally favorable; however, it yields lower nanomaterials, leading to less cost-effectiveness. Synthetic methods for producing nanomaterials via chemical and biological processes were compared (Saravanan et al., 2022). Nanomaterials yield, cost, environmental toxicity, and scalability are a few of the key parameters that can be used to compare the methods. Concerning the methods used, examples and case studies about their use in plant and soil-related nanomaterials synthesis could have been provided to show and justify the statement made in the conclusion section regarding which method is best for producing agri-nanomaterials (Ahmed et al., 2022). The integration of the forces for both chemical and biological synthesis of the mentioned processes of metal nanoparticles will be preferred (Singh R. P. et al., 2021).

### 3.3 Biological synthesis of nanomaterials

The physiochemical methodology has been the predominant way for formulating and synthesizing nanomaterials; nevertheless, utilizing biological techniques may provide supplementary benefits. Various NPs can be synthesized by integrating cyanobacteria, microalgae, actinomycetes, fungus, and yeast cultures with metals. The microorganism employed and many biosynthetic factors, including incubation duration, metal concentration, pH, temperature, and centrifugation, dictate the specific type of nanoparticle produced, varying from simple spheres to more intricate structures (Farda et al., 2024). The biological synthesis of nanomaterials facilitates precise control over their dimensions, morphology, and overall crystallinity, enhancing characterization efficacy. To attain optimal outcomes, it is imperative to choose the appropriate and most compatible nanomaterial for fabrication alongside the biofertilizer, as the characteristics of the final product significantly differ based on the type of nanomaterial employed (metal nano polymers, non-metal or metal oxide nano polymers, or carbon-derived nanopolymers) (Álvarez-Chimal and Ángel Arenas-Alatorre, 2023). Variability in nanomaterial, particle size, and composition will result in variation in fertilizer efficiency. It has an essential role in the adsorption and release kinetics, stability of the nanobiofertilizer, and plant uptake (Gade et al., 2023). Four major biological mechanisms include bacteria, algae, fungi, and plants, each having different advantages and challenges (Kulkarni et al., 2023).

#### 3.3.1 Bacteria

Microorganisms produce NPs through two distinct processes: external and internal synthesis. The specific process is contingent upon the type and configuration of the required NPs (Farda et al., 2024). Various microbes can transform inorganic substances into NPs via extracellular or intracellular mechanisms, wherein they assimilate metal ions from their media or environment and enzymatically decrease them to their elemental states. The preference of bacteria over the rest of the microorganisms for use is linked to their ease of growth in the laboratory and their inherent growth rate (Lahiri et al., 2021). When subjected to reactive ions in their environment, bacterial cells demonstrate an exceptional defense mechanism by transforming them into stable atoms, producing NPs (Pande et al., 2022). The other major constraint relates to the potential destruction of the bacterial cells throughout the synthesis course, besides environmental factors such as pH, temperature, and pressure affecting the yield (Kulkarni et al., 2023).

The extracellular synthesis of NPs involves several steps. Bacteria are usually grown in nutrient-rich media. After incubation, cells in the broth are removed (e.g., by centrifugation), and the cell-free broth containing the enzymes is treated with reductase enzymes and exposed to metal ions (Campaña et al., 2023). In contrast, the intracellular production of NPs utilizes the cellular mechanisms of microorganisms. The microbial biomass is cleaned using centrifugation to create a biomass pellet, thereafter washed with sterile distilled water in an appropriate liquid medium (Nyabadza et al., 2023). A metal solution in water is then introduced to the microbial biomass. Specific incubation conditions must be utilized for culturing a mixture of metals and microbial biomass until significant color changes are seen. The appearance of diverse colors indicates the formation of NPs. The production of zinc and manganese NPs is characterized by a whitish-yellow to yellow hue, gold NPs by a light yellow to pinkish tint, and silver NPs by a pale yellow to brownish coloration (Ibrahim et al., 2024). The negatively charged microbial cell walls uptake positively charged metal ions during this internal process. The metal ions experience enzymatic bioreduction within the cell wall, forming nanoclusters that allow NPs to diffuse into the solution from the cell wall. Accordingly, microbial cells took up the metal ions during intracellular synthesis and were subsequently converted into NPs inside the cell with the help of cellular enzymes. In the case of extracellular synthesis, the metal ions are released outside the cell surface and then reduced by cellular enzymes to form NPs (Koul et al., 2021).

The bacterial cells proved to be efficient bionanofactories in the production of different kinds of metallic NPs, for example, silver (Ag), gold (Au), copper (Cu), selenium (Se), and iron (Fe). Besides, they have been used to produce different kinds of metal oxides such as silver oxide (Ag_2_O), copper oxide (CuO), zinc oxide (ZnO), titanium oxide (TiO_2_), manganese oxide (MnO_2_), magnesium oxide (MgO), and iron oxide (Fe2O3) (Ibrahim et al., 2024).

#### 3.3.2 Fungi

Fungi provide major developmental production of NPs owing to their efficiency in capability for the release of extracellular enzymes. This will involve enzymatic reduction of metal ions, thereby leading to the end product nanoparticle formations. Their role in the synthesis of NPs also offers economic benefits and helps in biomass management (Momeni and Nabipour, 2015). While that is progress, issues regarding genetic modification during the biosynthesis process are considered, with lower production rates using NPs. Continuing investigations consider those issues, attempting to enhance efficiency and standardize fungal-mediated nanoparticle manufacturing. Producing NPs with fungus and yeast is a practical biological approach (Siddiqi and Husen, 2016). Due to their significant cell wall-binding capacity with metal ions and resilience to metal concentrations, fungi are exceptionally proficient at synthesizing NPs. Fungi-based biosynthesis is economically advantageous, and downstream processing is less complex than bacteria. The extracellular synthesis of NPs utilizing fungus is favored to eliminate the need for detergents and physical agents (Šebesta et al., 2022). The factors affecting nanoparticle yield and size are medium pH, reaction duration, and ionic concentration. Diverse fungal strains have been investigated for producing several metals and metal oxide NPs. The fungi *Trichoderma viride* and *Hypocrea lixiviate* have also been utilized to synthesize gold and silver NPs extracellularly. The size and yield depend on pH, temperature, and incubation time. Synthesis of silver NPs with good antimicrobial activity has also been done with yeasts like *Yarrowia lipolytica* and *Candida utilis* (Loshchinina et al., 2023). Silver NPs synthesized by the genetically modified yeast *Pichia pastoris* were stable and consistent in size. Insecticidal and mycogenic CuO NPs enhance wheat seed germination. Biosynthesis of ZnO NPs involves biosynthesis by *Aspergillus terreus*, *Xylaria acuta*, and some more fungi. These biosynthesized ZnO NPs work as an antimicrobial agent and also as an anticancer agent. Zinc oxide and copper oxide NPs serve as potent antibiofilm and antibacterial agents against multidrug-resistant microorganisms (Uthra et al., 2024). Titanium dioxide (TiO_2_) NPs synthesized with Baker’s yeast and Pleurotus jammer have photocatalytic, anticancer, and antibacterial properties. Iron oxide NPs (IONPs) produced by *Aspergillus japonicas* and caulicolous fungi have stable chemisorption characteristics, indicating possible biomedicine and water remediation uses (Rathore et al., 2023).

#### 3.3.3 Algae and plants

The biogenesis of NPs by algae is based on their native metabolic diversity and ability to grow well under various environmental conditions (Chaudhary et al., 2020). As autotrophic organisms, algae are unique “nano factories” that produce various NPs. Algae possess biologically active compounds and secondary metabolites as capping agents in nanoparticle production (El-Sheekh et al., 2023). Algae act like nature’s nanoengineers, enabling the improved synthesis of NPs with various properties. Advantages of algae-mediated synthesis include environmental compatibility, scalability, and the possibility of fabricating NPs with designed properties (Chaudhary et al., 2020).

Green synthesis using plant extracts boasts many advantages: it provides a non-contaminating approach to synthesizing NPs; different plants provide many secondary metabolites to facilitate reduction reactions during the formation of NPs. This current methodology reduces the risk of contamination while increasing the rates of reactions, maintaining cellular structural integrity (Shah et al., 2015). Plants and their components have been thoroughly investigated for the creation of NPs. The method’s simplicity, cost-effectiveness, and environmental sustainability instill reassurance and trust in its potential. Green synthesis utilizing plant extracts, including neem leaves, green tea, and other fruit extracts, is a simple and scalable method for the production of NPs (Marouzi et al., 2021).

## 4 Nanotechnology and its agricultural applications

Despite its nascent application in plants and ongoing investigations into its mechanisms of action, research demonstrates that nanotechnology influences numerous facets of plant life, such as enhancement of growth and resilience to diverse environmental challenges (Hayat et al., 2023). Nanomaterials are used as nano pesticides, herbicides, fertilizers, protective coatings, and nano-based devices to improve and monitor agricultural productivity. Metal-based nanomaterials, recognized for their antimicrobial characteristics, are especially efficacious against bacteria and fungi. The extensive antimicrobial efficacy of silver NPs against fungi, bacteria, and viruses is well-established (Shelar et al., 2023). These NPs impede the proliferation and establishment of pathogens, decreasing plant disease occurrence and mitigating yield losses. Nanomaterials’ seed coverings protect against pests and diseases and can boost plant defense mechanisms, generating immune responses resulting in systemic acquired resistance and enduring protection against pathogens (Selvakesavan et al., 2023; Shelar et al., 2023). Nanomaterials provide diverse approaches for alleviating abiotic stress by augmenting the mechanical integrity of essential plant structures, promoting adequate hydration, and boosting nutrient management efficiency (Ditta and Arshad, 2016). Nanomaterials also act as nano-carriers that protect the active molecule, enhance stability, and improve dispersion with specific delivery (Law et al., 2023). Such substances, microorganisms, and nanomaterials create a synergistic effect that has effectively relieved plants’ biotic and abiotic stresses (Jeon et al., 2024). With enhanced resilience and productivity, this technique promises to revolutionize agricultural practices. Ongoing study and deliberate implementation of nanotechnology can facilitate a more robust and sustainable agriculture. The following sections comprehensively analyze the many tactics employed to achieve these objectives.

### 4.1 Nano-technology in seed germination improvement

Material types such as carbon nanotubes, silicon dioxide, zinc oxide, titanium dioxide, and gold NPs positively affect seed germination. The stimulation of seed germination largely depends on genetic nature, environmental conditions, access to water, and land productivity. Metal NPs offer an enhanced surface area for electron transfer with biomolecules (Lahiani et al., 2013). Non-metallic NPs, including multi-walled carbon nanotubes, enhance the seed’s water absorption capacity by infiltrating the seed coat, stimulating enzymatic metabolism, and ultimately facilitating seed germination and seedling development in diverse crops (Li et al., 2021).

The OH-functionalized carbon nanomaterials, fullerenes, were found to induce a promising effect on plant growth promotion. In *Arabidopsis*, the impacts of fullerenol increased cell division, thereby enhancing the development of hypocotyl. Fullerol-based treatments for plant seeds increased the content of bioactive molecules (i.e., cucurbitacin-B and lycopene) and development (i.e., number, size, and yield of fruits) compared to a control of *Momordica charantia* (Husen and Siddiqi, 2014).

### 4.2 Nanotechnology to improve plant growth

NPs have demonstrated potential in sustainable agriculture as agents for plant growth (Karnwal et al., 2023). Bioinoculants utilizing NPs can improve nutrient absorption by functioning as transporters or slow-release fertilizer systems (Qiu et al., 2022; Tymoszuk et al., 2022), yields, and food production (Daniel et al., 2022). They can enhance soil structure, promote beneficial microbial communities, and diminish the necessity for chemical inputs (Majumder et al., 2023). Furthermore, they can facilitate soil restoration and remediation efforts by decreasing soil erosion, enhancing water retention, and mitigating the detrimental impacts of pollutants (Majumder et al., 2023; Vinzant et al., 2023). They further improve the health of plants and soil, photosynthesis, nutrient uptake, and microbial diversity (Agri et al., 2022). These nanoparticle-based bioinoculants also have several advantages: they are cost-effective, eco-friendly, act by some definite mode of action, and do not emit greenhouse gases (Alharbi et al., 2022). Besides, the amalgamated action of diverse microorganisms and nanomaterials in nanoparticle-based bioinoculants could improve crop productivity related to growing populations, biotic-abiotic stressors, soil health, and reduced use of chemical fertilizers (Chaudhary et al., 2021).

Table 2 presents various applications of nanomaterial-based bioinoculants in agricultural practices, specifically focusing on different types of NPs and their effects on plant growth and development (Karunakaran et al., 2013; Kole et al., 2013; Pallavi Mehta et al., 2016; Nawaz and Bano, 2020; Ma et al., 2022; Salama et al., 2022; Azarin et al., 2023; Karnwal et al., 2023). Nanomaterial-based bioinoculants contain NPs in variable types, like carbon-based, zinc oxide, ferric oxide, silica, and silver. These NPs improve plant characteristics such as biomass production, yields, chlorophyll content, and antioxidant activities through enhanced nutrient uptake, seed germination rates, and tolerance to stress. Applications modes are variable; these include soil and foliar applications. Generally, the prescribed concentration was low. The combination of NPs with biofertilizers or organic materials furthers the performance of plants. Although they have some advantages such as improvement in the quality of crops and reduced use of chemical fertilizers, their management should be done with care to avoid environmental hazards and toxicity issues.

**Table 2:** A comparison of the different preparation of nano materials methods.

Method category	Techniques	Description	Advantages	Disadvantages	Environmental impact	References
Physical	Flame Pyrolysis, Ball Milling, Laser Ablation	Physical methods involve processes such as flame pyrolysis, ball milling, and laser ablation. These methods use physical forces (thermal, mechanical, or laser energy) to produce nanoparticles.	Suitable for large-scale production; requires easily available equipment.	High energy consumption; difficulty in achieving low-cost, large-scale production.	High energy consumption, but no toxic reagents.	El-Khawaga et al. (2023) and Haris et al. (2023)
Chemical	Sol–Gel, Precipitation, Microemulsion, Hydrothermal-Solvothermal	Chemical methods rely on chemical transformations to synthesize nanoparticles. Examples include sol–gel, precipitation, microemulsion, and hydrothermal-solvothermal processes.	Simple, cost-competitive, and eco-friendly; provides control over particle size and morphology.	Energy-consuming and requires toxic reagents; limited scalability in large-scale production.	Use of toxic reagents (e.g., hydrazine, cyanides) affects environmental safety.	Navas et al. (2021) and Jabbar et al. (2022)
Biological	Green Synthesis, Enzymatic Synthesis, Microbial Synthesis	Biological methods involve the use of enzymes, microorganisms, or plant extracts for the synthesis of nanoparticles. These methods are eco-friendly but time-consuming and difficult to scale up.	Eco-friendly and non-toxic; compatible with agricultural applications.	Time-consuming; high cost of proteins/enzymes and limited scalability.	Eco-friendly due to the use of natural extracts or biological organisms.	Ahmed et al. (2022) and Hu et al. (2024)

### 4.3 Nanotechnology for mediating biotic and abiotic stress tolerance

Plant and post-harvest diseases induced by biotic stresses necessitate a departure from conventional agricultural techniques and pesticide application (Ansari, 2023). Nanotechnology can mitigate the phytotoxicity of certain pesticides, decreasing their concentrations while improving their delivery (Pérez-de-Luque and Rubiales, 2009). For example, a recent study showed that Mo-based nano-fungicides prevent the growth of conidial and conidiophores in plants and disrupt the mycelium or vegetative part of the fungus (Salama et al., 2022). Copper oxide NPs were applied to reduced graphene oxide nanosheets to create nanocomposites, which were subsequently utilized to investigate the plant pathogen *Fusarium oxysporum* in tomato and pepper plants. The fungicide Kocide 2000 (2.5 g/L), which mitigated the severity of *Fusarium* wilt and root rot disease through inducing cell death, was less efficacious against fungi than the nanocomposite, which, at 1 mg/L, exhibited superior antifungal activity by generating a more significant number of pits and perforations on the fungal cell membranes (El-Abeid et al., 2020). Nanotechnology can also be used to substitute pesticides. For example, it has been proved that tomato bacterial wilt occurs less frequently with the application of metal oxide NPs (CuO) due to their alteration of the structure and composition of the rhizospheric bacterial community (Sujitha et al., 2017).

To enhance plant tolerance to abiotic stress, NPs primarily augment the activity of antioxidant enzymes and eliminate reactive oxygen species (ROS). For example, foliar application of ZnO NPs enhanced chlorophyll content and leaf dry weight in cucumber plants (Singh D. et al., 2021). Compared to the untreated control group, the treated cucumber leaves exhibited a marked enhancement in the activity of antioxidant-related enzymes, including catalase and superoxide dismutase (Li et al., 2021; Sharma et al., 2023). When cerium ions interact with hydroxyl radicals, superoxide anions, and hydrogen peroxide, they can produce neutral molecules, including oxygen, water, and hydroxide ions. By enhancing photosynthesis and offering photoprotection, NPs modify the architecture of plant cells and increase the number of proteins and organic molecules (Wu et al., 2017). Nanomaterials have shown the potential to enhance plant growth and resistance to various abiotic stresses like salinity, drought, heavy metal toxicity, and extreme temperature fluctuations by nanomaterials to enhance plants’ growth, tolerance, and productivity (Thabet and Alqudah, 2024). These phenomena could be attributed to modulation of reactive oxygen species, change in gene expression, and improvement in physiological processes such as photosynthesis and water absorption (Xiong et al., 2021).

Customized NPs help to increase pesticide effective absorption and help the crops cope with adverse conditions without affecting yield and nutritional quality. Modifying microbial activity and enhancing their population also enhances the bioavailability of nitrogen and phosphorus (Simonin et al., 2018). Researchers have demonstrated that NPs induce stomatal closure and enhance AtGALK2 gene expression in *Arabidopsis* plants, augmenting their resilience to water stress (Camara et al., 2019). FeSO_4_ NPs significantly augmented chlorophyll pigment production (36–57%), raised shoot organic matter, accelerated CO_2_ assimilation rates, enlarged leaf area, optimized photosystem II functionality, elevated iron content, and resulted in a pronounced (15%) reduction in leaf salt content. Due to their antioxidant capabilities, CeO_2_ NPs augmented the photosynthetic rate by 67% under saline and high-temperature environments, improving agricultural productivity (Ansari et al., 2023). Nano-technology has facilitated the enhancement of food quality and availability and the development of vital agricultural products, particularly by optimizing the utilization of essential nutrients from various agrochemicals and improving crop productivity through superior pest and nutrient management integration (Ansari et al., 2023). Additionally, recent studies addressed the potential xenobiotic chemicals presented by NPs by applying sustainable production processes and exploring straightforward, rapid, and cost-effective bio-emerging mechanisms for the degradation and elimination of potentially harmful substances (Yadav et al., 2023).

### 4.4 Role of nanomaterials in reducing abiotic and biotic stresses

Nanomaterials have developed as promising materials to cope with different abiotic and biotic stresses. Nanomaterials can counteract abiotic stresses by altering antioxidant enzymes and preserving water in plant tissues (Zia-ur-Rehman et al., 2023). Further enhancement of the mechanism involves the close dermis culture system between the soil and the nanomaterials, making a straightforward approach for nutrient acquisition and protection against excessive absorption, making it a nutrient-rich agricultural commodity. By improving stress resistance, nanomaterials can contribute to food, feed, seed production, and sustainability. Nanomaterials may effectively combat biotic stresses (Zain et al., 2023). Most of the recent scientific reports on nanoparticle-nanoformulation interaction with plants focus on environmental stressors, adversities, and abiotic stressors. However, new findings suggest that nanomaterials can counteract biotic stresses. The mechanisms of regulation need to be better understood and need to be addressed (Priya et al., 2023). Nanomaterial-biotic stress interactions with plants have been reported for complex multitrophic systems with herbivores or pathogens, and the mechanisms appear to be homologous between nanomaterials in environmental and agricultural systems. Usually, nanomaterials provide antioxidants, react with the plant immune system to strengthen resistance, distinguish biotic and abiotic stress response signaling pathways, and suppress key target gene expression to assist in stress factors (Munir et al., 2023). Nanomaterial-biotic stress interactions have not been closely studied in nano agrochemistry, and it is unclear whether any of the reported use cases involve actions against plant disease or leaf injury. Because complex host-pathogen interactions have been investigated for so long, investigating the complex interactions of nanomaterials with abiotic and biotic stresses will likely take years or decades (Yadav et al., 2023). Nevertheless, such research will determine the probable practical applications and limitations that need to be addressed to avoid problems in the field. Furthermore, it is feasible that data generation interactions result in a reduction of biotic and abiotic stresses and a simultaneous enhancement of plant health (Guzmán et al., 2022).

### 4.5 Case studies of successful implementation

Many innovative initiatives have been carried out that show that nanomaterials can very well be used in agriculture. These successful case studies show the potential of this material. The positive impacts on animal and plant health have been highlighted. The implementation of nanomaterials in different domains for tea, potatoes, and leaf tobacco has also been demonstrated. The help to increase productivity and use of resources has been shown according to the choice of type of molecules for formulation and application (Zhao et al., 2020). Nanofertilizers have also been shown to save labor, water, and energy. However, successful cases are reviewed and discussed to provide different visions of using nanomaterials in agriculture. Various approaches and the use of basic and advanced technologies have been presented for achieving significant improvement in growth parameters, controlling, among others, rots, pests, and diseases, and avoiding a low number of active molecules concerning conventional approaches (Babu et al., 2004). One of the most important results of these case studies is the economic value, quantified in terms of the percentage of yield increases, reduced amounts of active compounds, support of parameters such as resistance to mechanical injury, lower percentage of grubs in treated plots, weed biomass, crop corm, etc., concerning the crop of reference. This economic value is always positive, ranging from −15 to +55%. Some interviews reported a decrease in the effect of nanomaterials of approximately −20%, while others reported an increase of +80% (Kaur and Kaur, 2021).

## 5 Microbe–nanoparticle interaction in plant stresses counteraction

### 5.1 Synergistic effect of microorganisms and NPs on plants

**Figure 1:**
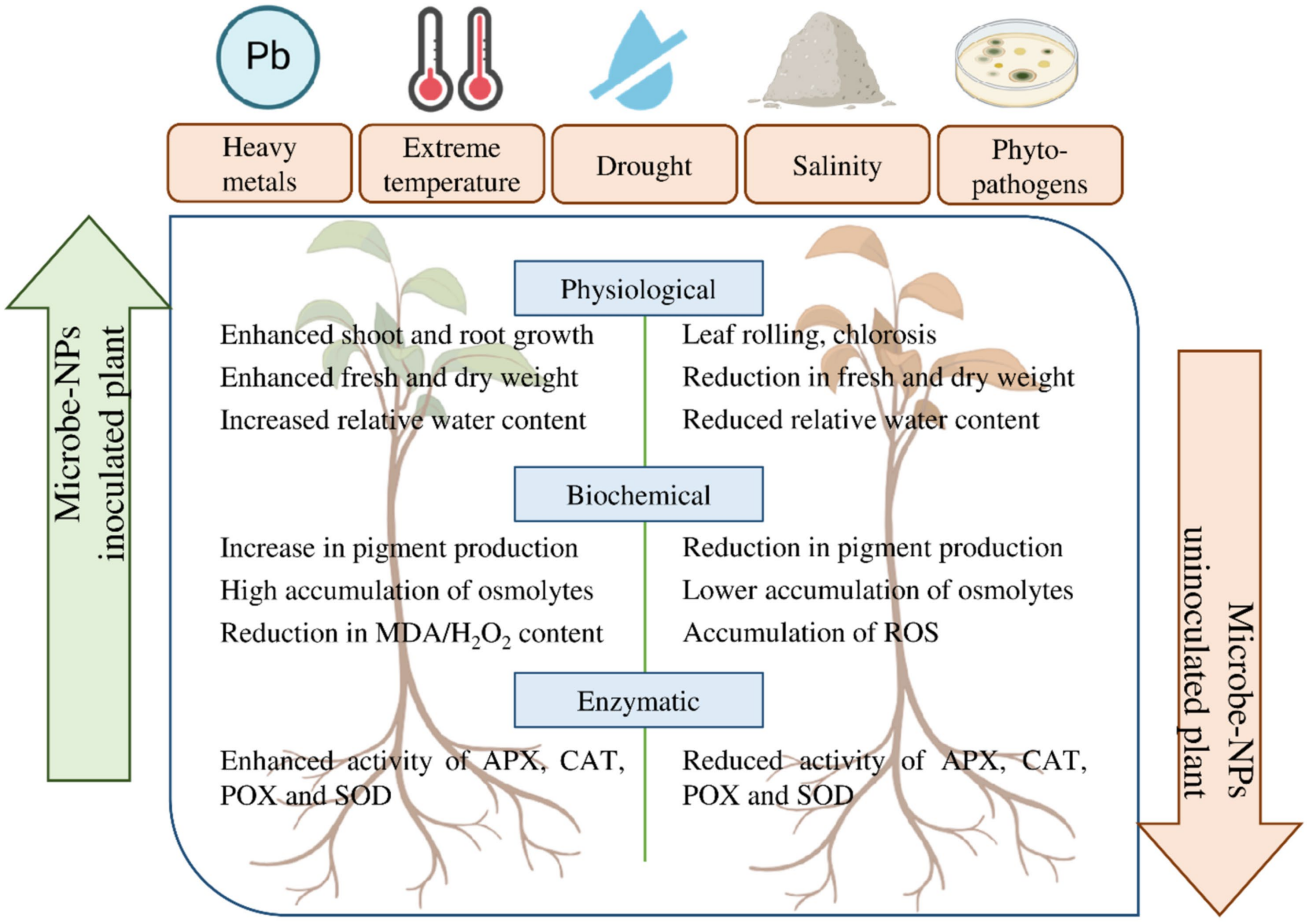
Synergistic effect of microbe-nanoparticles on physiological, biochemical and enzymatic attributes of plants under biotic and abiotic stresses (Where, NPs, nanoparticles; APX, ascorbate peroxidase; CAT, catalase; POX, peroxidase; SOD, superoxide dismutase).

Nanoparticle interactions happen through a broad set of processes, including, but not limited to, electrostatic interactions, chemical bonding, and biosorption. Integrating nanotechnology and microbiology in agriculture has garnered significant attention in recent years, potentially enhancing crop productivity, resistance, and resilience (Figure 1). This interaction assists nutrient acquisition and synthesizes growth-promoting phytohormones that govern plant activities. In a stressful environment, such microbes confer a supplementary benefit to plants (Naamala and Smith, 2020; Akhtar et al., 2021). Microbes-nanoparticle interaction can enhance nutrient delivery to plants encapsulated in NPs, particularly under adverse conditions. Furthermore, the gradual and consistent release of advantageous chemicals enhances the likelihood of plant survival (Szopa et al., 2022).

Table 3 presents information on the use of various NPs synthesized by microbes to enhance plant stress responses. The survey of these studies underlined that different NPs synthesized by microbes can alleviate various plant stressors, leading to improved growth, enhanced defense mechanisms, and better yields (Hossain et al., 2019; Ibrahim et al., 2020; El-Saadony et al., 2021b; El-Saadony et al., 2021a; Joshi et al., 2021; Manzoor et al., 2021; Priyanka et al., 2021; Singh, 2021). The capacity of microbe-based NPs to transform plant protection tactics is a noteworthy discovery. The initiation of systemic resistance in plants, a multifaceted process that engages numerous signaling pathways, is affected by the physicochemical characteristics of NPs, which can directly suppress certain plant diseases (Campos et al., 2023; Tortella et al., 2023) (Tables 4–6).

**Table 3:** Examples of nano-synthesis by bacteria, fungi, and plants.

Biological agent type	Organism/Material	Nanoparticle synthesized	Application target	Reference
Bacteria	*Bacillus megterium*	Selenium nanoparticles	Control of *Rhizoctonia solani* in Faba been	Hashem et al. (2021)
Fungi	*Aspergillus Niger*	Silica nanoparticles	Control of *Rhizoctonia* in egg plant	Albalawi et al. (2022)
Plant	Ziziphus spina Christi-extract	Silver nanoparticles	Control of *Fusarium oxysporum* in pepper	Abdelaziz et al. (2023a)
Plant	Clove essential oil	Nano emulsion	Control of *Fusarium neoscytalidium* in *Carum carvi*	Hashem et al. (2023)
Plant	Thyme essential oil	Nano emulsion	Control of *Fusarium* wilt in *Foeniculum vulgare*	Attia et al. (2023)
Plant derived	Gum Arabic	Bi mettallic ZnO CuO	Control of *Alternaria solani* in Potato	El-Batal et al. (2023)

**Table 4:** List of nanomaterial based bioinoculants for the plant growth and promotion.

**Nanoparticles**	**Impact of nanomaterial based bioinoculants**	**Amount applied**	**Inoculation approach**	**References**
Carbon-based fullerol NPs	Carbon-based fullerol NPs	0.943–47.2 nm	Soil	Kole et al. (2013)
Biofertilizer (*Rhizobium*), organic fertilizer and zinc NPs	increased plant height, leaf number, and biomass leaf area Increased protein, carbohydrate, and nutrient absorption in *Phaseolus vulgaris*	50, 100mgkg − 1	Soil	Salama et al. (2022)
Biofertilizer (composted biochar farmyard manure), and Zinc NPs	Enhanced antioxidants, biomass, yield, and photosynthetic pigments in plants *Triticum vulgare*	20 mg/g off soil	Soil and foliar	Pallavi Mehta et al. (2016)
nTiO_2_-activated carbon composite	In tomato and mung bean plants, the germination of seeds can be sped up and the germination time shortened by using the right amount of NP.	0–500 ppm	Foliar	Ma et al. (2022)
Nano-Zinc oxide particles	Greatly enhanced leaf gas exchange, chlorophyll content, osmolytes, metabolite profile, antioxidative enzyme activity, and plant development in *Hordeum vulgare*	12–250 ppm	Soil	Azarin et al. (2023)
Nano Ferric oxide particles	Higher leaf biomass and seed weight in *Glycine max*.	0.25–1 M	Foliar	Karnwal et al. (2023)
	In *Sorghum bicolor*, NPs improve plant performance and yield component, improve grain nutritional profile, and increase N and K element uptake.	6 mg/Kg	Soil and Foliar	
Biofertilizer (compost and biochar) with ferric NPs	Plant height and growth are enhanced in *Brassica juncea*, further pH, total nitrogen, phosphorus, and carbon all increased in soil.	50, 100mgkg − 1	Soil	Salama et al. (2022)
Silica NPs	Increased the overall concentration of NPK (nitrogen, phosphate, and potassium), which allowed all of the maize seeds in *Zea mays* to germinate.	150–300 ppm	Soil	Karunakaran et al. (2013)
Ag- NPs	Increased chlorophyll content, photosynthetic activity, biomass, and plant development significantly. Overall, *Cucumis sativus* has an enhanced endogenous antioxidant defense system.	1–5 mg/plant	Foliar	Nawaz and Bano (2020)

**Table 5:** Biotic stress management using nanotechnology.

Nanomaterial/Nanotechnology	Mechanism of action	Targeted biotic stress	Reference
Nano-silver (AgNPs)	Antimicrobial activity through the generation of reactive oxygen species (ROS) and disruption of microbial cell membranes.	Fungal and bacterial infections	Hajong et al. (2019)
Chitosan nanoparticles	Induce plant immune responses and act as carriers for bioactive compounds.	Viral and bacterial infections	Adetunji and Ugbenyen (2019)
Nano-encapsulated pesticides	Controlled release of active ingredients to improve pest control efficiency and reduce environmental impact.	Insect pests and pathogens	Adetunji and Ugbenyen (2019)
Silica nanoparticles	Provide a protective coating and strengthen the plant’s structural defenses.	Insect herbivory and fungal infections	Hajong et al. (2019)

**Table 6:** Abiotic stress management using nanotechnology.

Nanomaterial/Nanotechnology	Mechanism of action	Targeted abiotic Stress	Reference
Zinc oxide nanoparticles (ZnO NPs)	Regulate antioxidant enzyme activity, reduce ROS, and improve nutrient uptake.	Drought, salinity, and oxidative stress	Iqbal S. et al. (2020)
Iron oxide nanoparticles (Fe3O4 NPs)	Improve nutrient uptake and reduce heavy metal toxicity.	Heavy metal stress and salinity stress	Iqbal S. et al. (2020)
Carbon-based nanomaterials (e.g., carbon nanotubes, graphene oxide)	Improve water retention, enhance antioxidant activity, and regulate hormonal pathways.	Drought, salinity, and oxidative stress	Singh and Husen (2019)
Silicon nanoparticles (Si NPs)	Strengthen plant cell walls, reduce transpiration, and enhance antioxidant defense systems.	Heat, drought, and salinity stress	Jalil and Ansari (2019)
Gold nanoparticles (AuNPs)	Regulate photosynthesis, improve ROS scavenging, and stabilize enzyme activity.	Oxidative stress, drought, and temperature stress	Zhao et al. (2020)
Nanoclay	Acts as a moisture-retentive material, supporting soil water-holding capacity.	Drought stress	Singh and Husen (2019)

Additionally, other studies explored the suitability of this interaction to improve the effectiveness of plant growth-promoting bacteria-based biostimulants. For example, a biofertilizer using *Azospirillum brasilense* (strains AbV5 and AbV6) was developed with a chitosan nanomaterial. Maize plants treated with this nanotechnology resulted in a 19% increase in root length and a 17% improvement in shoot fresh weight. The chlorophyll b content in treated plants increased by 71%. The nanotechnology application also extended the survival of *A. brasilense* strains in the soil for at least 60 days (Lima-Tenório et al., 2023). Chitosan-immobilized silica nanocomposites (CISNC) containing *Glomus mosseae*, *Trichoderma viridae*, and *Bacillus subtilis* showed their efficacy against tomato bacterial wilt caused by *Ralstonia solanacearum* (Stasińska-Jakubas and Hawrylak-Nowak, 2022). The nanocomposites also improved water retention and enhanced physiological, biochemical, and soil microbial activity, boosting the efficiency of tomato yield and resource use. Nano biofertilizer formed by nano clay-encapsulated *Trichoderma* and *Pseudomonas* species induced resistance to fungal and nematode diseases and other abiotic stress factors in Rabi crops (Jiang et al., 2021) (Table 7).

**Table 7:** Abiotic and biotic stress mitigation using microbe-mediated NPs.

Nanoparticle	Microbe	Plant	Stress	Response	Reference
Silicon	*Aspergillus tubingensis*	Common bean	Salinity and heavy metal	High antioxidant enzyme activity and growth promotion of plant.	El-Saadony et al. (2021b)
Silver	*Pseudomonas rhodesiae*	Sweet potato	*Dickeya dadantii*	Growth inhibition of pathogen.	Hossain et al. (2019)
Silver	*Pseudomonas poae*	Wheat	*Fusarium graminearum*	Deformation of fungal hyphae inhibiting its growth.	Ibrahim et al. (2020)
Selenium	*Trichoderma atroviride*	Tomato	*Phytophthora infestans*	Activation of defense related enzymes and growth promotion of plant.	Joshi et al. (2021)
Iron oxide	*Pantoea ananatis*	Wheat	Cadmium and salinity	High plant biomass, antioxidant activity and pigment content.	Manzoor et al. (2021)
Copper	*Klebsiella pneumonia*	Corn	Salinity	Reduction in oxidative stress and growth promotion of plant.	Priyanka et al. (2021)
Zinc oxide	*Halimeda tuna*	Mexican cotton	Heavy metal	Enhanced physiochemical traits, pigment production and antioxidant defense.	Singh (2021)
Zinc oxide	*Aspergillus niger*	Potato	*Alternaria solani*	Reduced disease severity and increase in yield of plant.	El-Saadony et al. (2021b)

### 5.2 Molecular aspect of nanoparticle-mediated amelioration of stresses

Nanoparticles are a new material with specific physical and chemical properties; therefore, they can mitigate abiotic stresses in plants, including those caused by drought, salinity, and nutrient deficiency (Tortella et al., 2023). Stress tolerance in plants improves since important cellular processes like photosynthesis, water intake, and production of ROS are altered, thus maintaining their resiliency during conditions that are not favorable. Other molecular evidence shows that NPS controls the genes responsible for stress and metabolic pathways. For instance, Morteza et al., reported that the TiO₂ NPs improved chromatin assembly, photosynthetic efficiency, chloroplast protection, and chlorophyll content in tomatoes and wheat (Morteza et al., 2013). These NPs enhanced the photosynthesis yield, transpiration rates, and water conductivity. They trigger the cells to synthesize osmolytes, participate in developing phytonutrients, and enhance the biosynthesis of photopigments. Indeed, Sonkar et al., recorded some vital changes in morphology and biochemistry with responses to abiotic stresses (Sonkar et al., 2023). In the sub-cellular context, the nanoparticles interfere with the key enzymes and genetic machinery. Stress suppresses the activity of ribulose-1,5-bisphosphate carboxylase oxygenase RUBISCO and leads to degradation of the small subunit with the further reduction in CO₂ fixation rate. In contrast, NP acts inversely and transforms the gene expression. García-Sánchez et al. mentioned that under exposure to TiO₂ NPs, Ag NPs, and MWCNTs, there were 16 responsive genes to drought in *Arabidopsis thaliana* (García-Sánchez et al., 2015). Results regarding induced gene activity on chromatin, transcription, cell signaling, and response to cold stress were reported by Amini et al., under the exposition of TiO₂ NPs for *Cicer arietinum* (Amini et al., 2017). The essential outcome of these works underlined the molecular relationship between NPs and plant stress tolerance, showing how these NPs have been used in modulating genetic and biochemical pathways underpinning the resilience of plants. That would have defined targets and modes of action and thus allowed better efficiencies and sustainability for applications related to agriculture. Nanotechnology can also be considered a novel approach that may trigger innovations and might significantly affect modern agriculture. Nanotechnology for agricultural enhancement has greatly earned from the efficient transfer of genetic material, including DNA and RNA, through genetic engineering, gene editing, and gene cloning procedures (Ansari et al., 2021; Ansari, 2023). Gene transformation assisted by NPs is rapidly developing, enabling researchers to break through plant cell walls and membranes to surmount the inefficiencies of existing transgene delivery methods (Billingsley et al., 2020; Lv et al., 2020).

### 5.3 Impact use of nanotechnology in agriculture on the environment and biodiversity in agricultural soil

Considering global climate changes, nanotechnology is considered a transformational approach for increasing productivity and enhancing sustainability in agriculture. Using nanotechnology, novel solutions have been designed to solve serious agricultural problems concerning soil degradation, salinity stress, and severe weather conditions. So far, the most significant impact it has had has been in soil management. According to the findings reported by El-Ramady et al. (2024) and Jamal et al. (2024), nano-enabled soil amendment applications significantly improve soil structure, enhance nutrient availability, and stimulate microbial activity. Besides this, nanoparticles also act as slow-release fertilizers, providing an efficient mode of nutrient delivery, reducing runoff losses, and lowering environmental contamination. Applications of nanotechnology regarding soil salinity stress are similarly encouraging. According to El-Ramady et al. (2024), nanoparticles enhance the ion-exchange capacity and water retention of the soil, thus enhancing plant growth in saline conditions. Moreover, Shoukat et al. emphasize that nanoparticles protect plants from abiotic stressors, such as high temperatures, drought conditions, and contamination, and stand up to more vigorous climate-sensitive crop production systems (Shoukat et al., 2024). However, nanotechnology’s environmental and biodiversity impacts on agricultural soils are worth attention. In this respect, such influences may prove very dangerous for microbial communities responsible for nutrient cycling and decomposition of organic matter, according to Fernández-Triana et al. (2024). Properties and concentration of nanoparticles may favor microbial growth or inhibit the same. Therefore, agricultural activity can significantly affect the diversity and health of the soil. Efficient, sustainable nano-management requires an optimal balance to achieve the results (Sári et al., 2024), which puts into perspective strategies toward gaining maximum benefit from the application of nanotechnology with minimal ecological effects. Examples include controlled-release nanoparticles for agrochemicals that can reduce excessive use of fertilizers and pesticides, thus limiting pollution. Farming methods involving tiny materials, such as coatings applied to seeds, can guard against temperature changes and reduce dependence on artificial chemicals (El-Ramady et al., 2024). Finally, only a combined approach of nanotechnology and climate-smart farming strategies can develop a whole plan for sustainable food production (Sarma et al., 2024). The studies above show the great potential of tiny technology. It is supposed to be applied in climate-resilient agriculture. The development of future research on ecologically harmless nanomaterials should relate to long-term implications on soil biodiversity, ensuring the sustainability of safety from the nano-based methodology in agriculture. This development will be achieved by integrating nanotechnology into climate-smart agricultural strategies toward sustainable food production. These studies show the vast potential of nanotechnology. More future research needs to be directed toward developing innocuous, environmentally harmless nanomaterials with a long-term impact on soil biodiversity in tune with the sustainability and safety of nano-based agricultural methodology.

## 6 Conclusion

Abiotic and biotic stresses have become significant threats to the health of plants and agricultural productivity, further influenced by climate change. Plants have natural defenses against stresses; however, nontoxic sustainable inventions are required to enhance the stress tolerance of plants. Biologically synthesized NPs may work in a different direction; various studies have identified the effectiveness of the microbe-NP interaction in reducing stress in several crops. Studies on the synthesis, characterization, and application of NPs should be developed to achieve the benefits with minimum environmental safety. Green biodegradable NPs will contribute to sustainable agriculture by assuring their ecological compatibility. Advanced nanotechnology combined with omics -based knowledge may further lead to the creation of innovative nanomaterials targeted for particular stressors. That is how agronomy, microbiology, and nanotechnology together might revolutionize agriculture’s stress management for resilient, efficient, and sustainable farming.
